# The Chaperone Protein GRP78 Promotes Survival and Migration of Head and Neck Cancer After Direct Radiation Exposure and Extracellular Vesicle-Transfer

**DOI:** 10.3389/fonc.2022.842418

**Published:** 2022-03-01

**Authors:** Michael Schneider, Klaudia Winkler, Rosemarie Kell, Michael W. Pfaffl, Michael J. Atkinson, Simone Moertl

**Affiliations:** ^1^ Institute of Radiation Medicine, Helmholtz Zentrum München, German Research Center for Environmental Health, Neuherberg, Germany; ^2^ Animal Physiology and Immunology, TUM School of Life Science, Technical University of Munich, Freising, Germany; ^3^ Chair of Radiation Biology, TUM School of Medicine, Technical University of Munich, Munich, Germany; ^4^ Department of Effects and Risks of Ionising and Non-Ionising Radiation, Federal Office for Radiation Protection, Oberschleißheim, Germany

**Keywords:** ionizing radiation, extracellular vesicles, HNSCC, HSP70 heat-shock proteins, radiotherapy, cell migration, bystander effect, GRP78

## Abstract

**Background and Purpose:**

Increased levels of the chaperone protein GRP78 have been implicated in poorer outcomes of cancer therapy. We have therefore explored the functional connection between the expression of GRP78 and the development of radioresistance and metastatic behavior in HNSCC.

**Material and Methods:**

The association between gene expression of GRP78 and survival in HNSCC patients was examined using the TCGA database. The influence of ionizing radiation on the GRP78 levels in HNSCC cell lines, their secreted extracellular vesicles (EV) and non-irradiated EV-recipient cells was investigated by Western Blot and FACS. The consequences of chemical inhibition or experimental overexpression of GRP78 on radioresistance and migration of HNSCC cells were analyzed by clonogenic survival and gap closure assays.

**Results:**

Elevated levels of GRP78 RNA in HNSCC correlated with poorer overall survival. Radiation increased GRP78 protein expression on the surface of HNSCC cell lines. Experimental overexpression of GRP78 increased both radioresistance and migratory potential. Chemical inhibition of GRP78 impaired cell migration. EVs were identified as a potential source of increased GRP78 content as elevated levels of surface GRP78 were found in EVs released by irradiated cells. These vesicles transferred GRP78 to non-irradiated recipient cells during co-cultivation.

**Conclusions:**

We have identified the chaperone protein GRP78 as a potential driver of increased radioresistance and motility in HNSCC. The uptake of GRP78-rich EVs originating from irradiated cells may contribute to a poorer prognosis through bystander effects mediated by the transfer of GRP78 to non-irradiated cells. Therefore, we consider the chaperone protein GRP78 to be an attractive target for improving radiotherapy strategies.

## Introduction

Therapy options for head and neck squamous cell carcinomas (HNSCC) have improved over the last decades, tobacco consumption is declining and HPV-positive status is a good prognostic marker for tumor treatment. Yet especially HPV-negative and advanced-stage tumors still have a poor prognosis and an overall 5-year survival rate of approximately 60% (1, 2). HNSCC are highly invasive and metastatic and frequently acquire treatment resistance. This resistance results in an increase in treatment doses of radiation and chemotherapy, which in turn leads to worse off-target toxicity. Still, standard therapy protocols combine surgery with radiotherapy and chemoradiotherapy despite the severe side effects and low effectiveness (2, 3), especially in non-resectable or incompletely resected tumors (4). A better understanding of the molecular mechanisms responsible for this intransigence is strongly needed for new therapeutic strategies and to improve therapy outcome.

The 78-kDa glucose-regulated protein (GRP78/BiP), a molecular chaperone of the heat shock protein 70 family, participates in biological functions that are relevant to a poor response to cancer therapy, in particular the control of the unfolded protein response (UPR) *via* activation of transmembrane ER-stress sensor proteins and calcium storage (5, 6). In HNSCC and other tumor types, the UPR is constantly activated by numerous stressors of the tumor microenvironment such as hypoxia or nutrient deprivation (7, 8) leading to an upregulation of GRP78 expression in tumor cells which is considerably higher compared to non-malignant cells. Besides accumulating in the ER to counter ER stress, a function in tumor cell signaling and communication is suggested as GRP78 is translocated to the cell surface of tumor cells upon different cellular stressors such as hypoxia (9–11). Given that GRP78 has no transmembrane domain other proteins like Cripto are needed to interact with the chaperone and thereby connecting it to the cell surface (12, 13). This cell surface GRP78 is suggested to trigger tumorigenic and metastatic signaling and induce resistance to treatment in various cancer models, although the exact mechanisms are still unclear (14, 15).

A contribution to intercellular signaling has been suggested by the observation that GRP78 is present in extracellular vesicles (EVs) (16, 17). These small (50-1000 nm) vesicles, surrounded by a phospholipid bilayer, are released into extracellular fluids and contribute to cell-cell communication. The most important EVs are exosomes, with endocytic origin, and microvesicles, formed directly at the plasma membrane (18). In HNSCC, we have previously demonstrated that EVs released from irradiated cells are able to increase the motility and radioresistance of recipient cells and proteomic analyses showed increased vesicular levels of GRP78 (17, 19).

We now report that surface GRP78 expression is increased by exposure to ionizing radiation in two HNSCC cell lines. Increased GRP78 promotes radiation resistance and increases metastatic behavior of these cells. Moreover, irradiation increased the GRP78 content of released EVs, and these were able to transfer GRP78 to non-irradiated recipient cells. This increase in GRP78 may contribute to a bystander effect that increased radiation resistance and metastatic behavior in non-irradiated cells, suggesting that EV transfer of GRP78 may be responsible for some of the deleterious behavior and poorer prognosis of GRP78 overexpressing tumors.

## Materials and Methods

### TCGA Data Analysis

Differential expression analysis of HNSCC patient transcriptomes, taken from The Cancer Genome Atlas (TCGA), was performed using the R Studio software with packages ‘BiocManager’, ‘TCGAbiolinks’, ‘limma’ and ‘edgeR’ (20–24). A glmLRT fit was performed with FDR and logFC cut-offs set to 0.01 and 1, respectively. Kaplan-Meyer plot of patient survival analysis was generated using the Xena Functional Genomics Explorer (University of California, Santa Cruz) with default settings including a statistical analysis with log-rank test (25).

### Cell Culture

HPV-negative HNSCC cell lines BHY (DSMZ), FaDu (ATCC), CAL-33 (DSMZ) and SCC131 (DSMZ) were cultivated in a humidified atmosphere at 37°C and 5% CO_2_. DMEM (Dulbecco’s modified Eagle’s medium, Gibco) with GlutaMAX, pyruvate and high Glucose was used for BHY and CAL-33 cells. The same medium with low Glucose was used for FaDu cells. Minimal Essential Medium (MEM, Gibco) with GlutaMAX and Earle’s Salts was used for SCC131. The media were supplemented with 10% FBS (fetal bovine serum, GE Healthcare). Green Fluorescent Protein (GFP)-expressing derivatives of the cell lines BHY and FaDu (BHY-GFP and FaDu-GFP), generated as described previously (17), were cultivated in their respective media with additional 0.3 µg/ml or 0.1 µg/ml puromycin to maintain stable GFP expression.

Cell line identity was confirmed by genomic sequencing of nine marker loci: D5S818, D13S317, D7S820, D16S539, VWA, TH01, AM, TPOX, CSF1PO (Eurofins Genomics). Mycoplasma negative status was confirmed with MycoAlert (Lonza).

For GRP78 overexpression, 2.5 x 10^5^ cells were transfected with pcDNA3.1(+)-GRP78/BiP (32701, Addgene) or empty control vector (V79020, Invitrogen) using Lipofectamine 2000 Transfection Reagent (Invitrogen) according to the manufacturers protocol.

The GRP78 inhibitor HA15 (Selleck Chemicals) was diluted in DMSO with pure DMSO serving as control. FaDu and FaDu-GFP cells were treated with 1.5, 2.5 or 3.5 µM, BHY and BHY-GFP cells were treated with 20, 30 or 40 µM inhibitor.

Bovine EVs were removed from FBS by ultracentrifugation (100,000g, 4°C, 14h) to generate EV-depleted FBS (EdFBS).

### Irradiation

Cells were X-irradiated at room temperature with a dose rate of 0.82 Gy/min (Xstrahl RS225 X-ray system at 195 kV and 10 mA with a 3 mm aluminum filter). Sham irradiated cells were treated identically, without exposure.

### Protein Quantification and Immunoblotting

Cells were detached with 0.05% trypsin or collected with a cell scraper to retain surface proteins. Cell pellets were lysed at 4°C with T-PER lysis buffer (Thermo Fisher) combined with PhosSTOP and cOmplete, phosphatase and protease inhibitor cocktails (Roche). Protein concentrations were determined with the Pierce BCA Protein Assay Kit (Thermo Fisher).

For immunoblotting, either 10 µg protein or 15 µl of EV suspension were used. Primary antibodies used were Alix (2171, Cell Signaling), Beta-actin (SAB1305567, Sigma-Aldrich), Calnexin (sc11397, Santa Cruz), GAPDH (sc-47724, Santa Cruz), GRP78/BiP (3177, Cell Signaling), TSG101 (GTX70255, GeneTex) and CD9 (sc-13118, Santa Cruz). Proteins were revealed using horseradish-peroxidase conjugated secondary antibodies (anti-mouse: sc2005, anti-rabbit: sc2005; Santa Cruz) and the Amersham ECL Select Western Blotting detection reagent (GE Healthcare). Digital images were captured with the FluorChem HD2 (Alpha Innotec).

### Flow Cytometry

3 x 10^5^ seeded cells were harvested 24 h after irradiation with 6 and 0 Gy. After detaching with a cell scraper for intact surface proteins, cells were incubated with anti-GRP78 antibody followed by a secondary Alexa Fluor 488-coupled antibody (anti-rabbit: A-11008, Thermo Fisher). Incubation steps were alternated with PBS+0.5% bovine serum albumin washing steps. A minimum of 10,000 labelled cells were analyzed with a FACSCAN LSRII (Becton-Dickinson). To remove cellular fragments and dead cells from the analysis, cells incubated with secondary antibody only were used for gating according to their forward and sideward scatter properties. Afterwards, doublets were excluded by plotting FSC height vs. FSC area, remaining particles were analyzed in terms of fluorescence intensity and their mean intensity was documented and used for calculations.

For analysis of GRP78 expression on the EV-surface, 10 µl EVs (1 x 10^9^ EV/ml) derived from 0 or 6 Gy-irradiated BHY cells were absorbed onto 10 µl latex beads (A37304, Invitrogen) for 1 h and in 100 µl PBS overnight. Following 30 min incubation with PBS+1 M Glycine, cells were labelled with either anti-CD63 (sc15363, Santa Cruz) or anti-GRP78/BiP antibodies followed by Alexa Fluor 488-coupled secondary antibody. All incubation steps were performed at 4°C.

### Clonogenic Survival

Transfected FaDu and BHY cells (see section *Cell Culture*) were incubated for 48 h and seeded in 6-well plates at a range of cell densities. 24 h later, they were irradiated with different radiation doses ranging from 0 to 8 Gy and colonies were expanded for 10-14 days. Ethanol-fixed colonies were stained with Giemsa (1:10 in PBS, Boehringer Ingelheim) and counted. Survival fractions after 2 Gy-irradiation (SF2) were determined with R Studio.

### Cell Migration

Gap closure assays were performed using GFP-expressing cells following a previous protocol (17). 55,000 BHY or 75,000 FaDu cells were seeded into the individual wells of removable 12-well silicone grids (Ibidi), that were placed in 10-cm dishes. After 24 h, the silicone spacers were removed to create even gaps between the individual cell monolayers and 8 ml medium was added.

For inhibition of GRP78, the medium, added after removal of the silicone grids, contained HA15 or DMSO as control.

For overexpression of GRP78, the cells were seeded as stated above 48 h after transfection and normal medium was used.

Time-lapse images of the GFP-labelled cells were captured by fluorescence microscopy with a Biorevo BZ-9000 (Keyence). Migratory behavior was quantified from these images with the software package ‘countcolors’ in R Studio (26). A pixel intensity cut-off for green pixels of 20 (range: 0-255, RGB color space) was chosen based on the green pixel intensity of the 0h-background.

### EV Isolation and Characterization

1.25 x 10^6^ BHY cells, 1.5 x 10^6^ FaDu cells and 2 x 10^6^ CAL-33 or SCC131 cells were seeded with 8 ml medium in 10-cm dishes and irradiated with 0 and 6 Gy after 48 h. Prior to irradiation, the cells were washed with PBS and EdFBS-supplemented medium was added. 24 h after irradiation, small EVs were collected from the supernatant by serial ultracentrifugation and microfiltration based on the protocol by Mutschelknaus et al. (17). Two centrifugation steps of 300 g and 10,000 g (4°C) were followed by 0.22 µm-filtration. The filtrate was centrifuged three times at 100,000g (4°C) for 2 h. Resuspended EV pellet was stored at -20°C. EV size distributions were analyzed by NanoSight LM10 (Malvern).

### EV Transfer and Fluorescence Microscopy

For vesicle transfer, 3 x 10^5^ BHY or FaDu cells were seeded in 6-well plates. After 24 h, the wells were washed with PBS and 2 ml fresh medium containing 10% EdFBS was added, supplemented by the EVs isolated from 6 ml conditioned medium from 0 or 6 Gy-irradiated cells. 24 h later, cells were detached with a cell scraper and analyzed *via* Western Blot.

To confirm EV-uptake into recipient cells, the isolated EVs were stained with green fluorescent dye PKH67 (MINI67, Sigma-Aldrich) as previously described (19). 40,000 BHY cells were seeded in 12-well silicone grids (Ibidi) on glass slides, after 24 h the wells were washed with PBS and 250 µl new EdFBS-supplemented medium was added, containing PKH67-stained EVs isolated from 750 µl conditioned medium of BHY cells. After 24 h, cells were fixed in 4% paraformaldehyde and nuclei were stained with Hoechst 33342.

For visualization of surface GRP78 after irradiation, cells were seeded on glass slides and after 24 h irradiated with 0 and 6 Gy. Further 24 h later, cells were fixed with 4% paraformaldehyde and labelled with an anti-GRP78 antibody dilution of 1:100 (PA1-014A, Invitrogen) followed by a secondary Alexa Fluor 488-coupled antibody (anti-rabbit: A-11008, Thermo Fisher). Cells were not permeabilized for appropriate labelling of surface GRP78.

### Cell Viability Assay

Cell viability assay was performed with the PrestoBlue Cell Viability assay (Invitrogen) according to the manufacturers protocol.

For GRP78 inhibition, 5,000 cells were seeded in 96-well plates. After 24 h, medium was replaced with HA15- or DMSO-supplemented medium and cells were incubated for 24-72 h.

For GRP78 overexpression, 5,000 transfected cells (see *Radiation Increased Surface Expression of GRP78 in HNSCC Cell Lines*) were seeded in 96-well plates 48 h after transfection and incubated for 24-96 h.

### Statistical Analysis

Bioinformatic analyses including statistics were performed with R version 4.0.4 (27) and R Studio version 1.4.1106 (28). The package ‘ggplot2’ (29) was used for plots unless stated otherwise. For statistical analysis, the package ‘rstatix’ was used (30).

The data shown depict the mean +/- standard deviation of biological replicates (n). The significance level was chosen at 5%. For data with one factor variable, the paired, “two-sided” t-test was used and the Bonferroni correction was applied where appropriate. Remaining results were analyzed with 2-way repeated measures (RM) ANOVA and, when indicated, by pairwise “two-sided” t-tests with Bonferroni correction. Survival analysis was evaluated with log-rank test as mentioned in section 3.1.

## Results

### Increased GRP78 Gene Expression Is Associated With Worse Patient Survival

**Figure 1:**
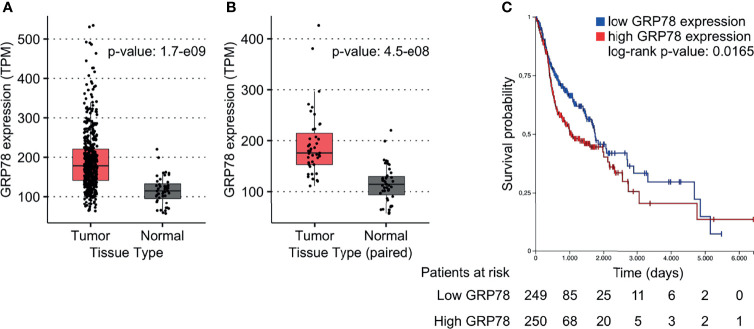
GRP78 expression and outcome in HNSCC patients. **(A, B)** DEA shows increased GRP78 gene expression in HNSCC tumor tissue (n = 500) in comparison to the non-cancerous tissue samples (n = 44). Data was retrieved from the TCGA database. **(B)** Comparison of GRP78 expression in paired tumor and non-cancerous tissue samples (n = 43 for both). **(C)** Kaplan-Meier (KM) survival analysis of HNSCC patients showing decreased survival associated with high GRP78 expression. Survival analysis was evaluated with log-rank test (p = 0.0165).

To investigate the effect of GRP78 on treatment outcome of HNSCC patients, the TCGA database was used. GRP78 gene expression in tumor samples from 500 HNSCC patients was significantly higher than that in non-cancerous tissue samples obtained from 44 patients (**Figure 1A**). Analysis of the 43 available pairs of tumor and normal tissue samples confirmed this difference (**Figure 1B**). For survival analysis, data from 499 patients were divided into groups with low and high GRP78 gene expression, with the expression median chosen as the separation point. The results for the Kaplan-Meier analysis of survival revealed that patients with GRP78 RNA levels above the median had significantly shorter overall survival than patients with GRP78 RNA expression below the median (**Figure 1C**).

### Radiation Increased Surface Expression of GRP78 in HNSCC Cell Lines

**Figure 2:**
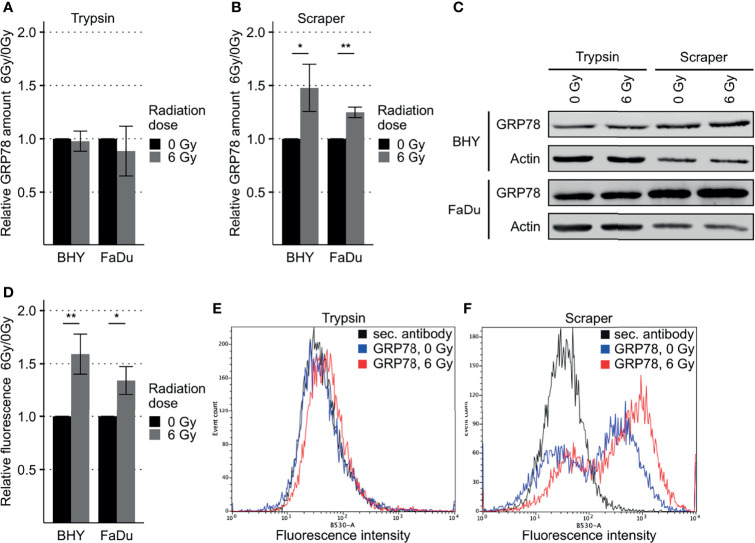
Increased GRP78 expression on the surface of irradiated HNSCC cells. **(A–C)** Western Blot analysis of GRP78 expression in non-irradiated (0 Gy) and irradiated (6 Gy) HNSCC cells harvested with trypsin show no difference **(A)**. Cells harvested with a cell scraper show an increase in GRP78 after irradiation **(B)**. GRP78 expression was normalized to beta-actin and 0 Gy samples served as baseline (n = 4, t-test, *p < 0.05, **p < 0.01). **(C)** Representative Western Blot images of GRP78 expression in BHY and FaDu cells. Beta-actin was used as loading control. **(D)** Flow cytometry analysis of surface GRP78 in 0 Gy and 6 Gy-irradiated HNSCC cells confirmed increased GRP78 expression after irradiation. Samples were normalized to secondary antibody signal and 0 Gy (n = 4, t-test, *p < 0.05, **p < 0.01). **(E, F)** Example histograms of GRP78 fluorescence intensity from BHY cells harvested with either Trypsin **(E)**, showing no difference in intensity, or a cell scraper **(F)**, showing an increase of signal intensity in irradiated cells.

GRP78 protein expression in response to radiation was monitored in BHY and FaDu HNSCC cell lines. Neither of the irradiated cell lines revealed a change in GRP78 content when cells were detached with trypsin (**Figures 2A, C**). However, when cells were harvested by scraping, leaving surface proteins intact, both irradiated cell lines showed a significant increase in GRP78 expression (**Figures 2B, C**). Whilst radiation increased the trypsin sensitive pool of GRP78 on the surface of both BHY and FaDu cells, the amount of trypsin resistant intracellular GRP78 did not change The same results could be observed in the HNSCC cell lines CAL-33 and SCC131 (**Supplementary Figures S1A–C**).

To confirm the increase in cell surface expression, the scraped cells were analyzed by flow cytometry without permeabilization. This analysis corroborated the western blotting by showing a significant increase in antibody-accessible (surface) GRP78 expression in irradiated cells in comparison to the non-irradiated control cells (**Figures 2D, F** and **Supplementary Figure S1D**). Surface expression in cells gathered by trypsinization was not detectable (**Figure 2E**). In addition, increased surface expression of GRP78 after irradiation of BHY cells could also be shown with fluorescence microscopy (**Supplementary Figure S2**).

### Overexpression of GRP78 Enhanced Radioresistance and Migration in HNSCC Cells

**Figure 3:**
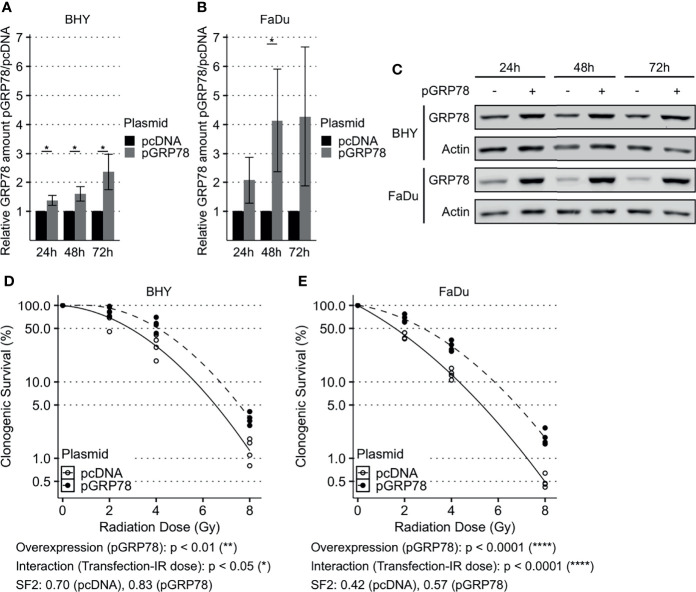
Enhanced survival of irradiated HNSCC cells after overexpression of GRP78. **(A–C)** Western Blot analysis of GRP78 shows increased expression in BHY **(A)** and FaDu **(B)** cells 24 h, 48 h and 72 h after transfection with GRP78 plasmid (pGRP78). GRP78 expression was normalized to beta-actin and control transfection (pcDNA) served as baseline (n = 4, 2-way RM ANOVA, *p adj. < 0.05). **(C)** Representative Western Blot images of GRP78 expression in pGRP78- and pcDNA-transfected BHY and FaDu cells. Beta-actin was used as loading control. **(D, E)** Clonogenic survival assay of BHY **(D)** and FaDu **(E)** cells exposed to radiation doses of 0, 2, 4 or 8 Gy 72 h after GRP78 transfection. Both cell lines show increased survival in GRP78 overexpressing cells. Values were normalized to 0 Gy control. Logarithmic scale was used for the relative cell survival (n = 4, Two-way RM ANOVA).

GRP78 expression of both BHY and FaDu cells was significantly increased 48 and 72 h after transfection with the expression vector, versus cells transfected with empty control vector (pcDNA, **Figures 3A–C**). Functional analyses were performed 72 h after transfection as GRP78 overexpression was higher at this timepoint.

To examine radioresistance, a clonogenic survival assay was performed. There was a significant increase in the radiation resistance of BHY and FaDu cells overexpressing GRP78 in comparison to vector control cells, embodied by a significant interaction parameter and an increase in the SF2 (**Figures 3D, E**).

**Figure 4:**
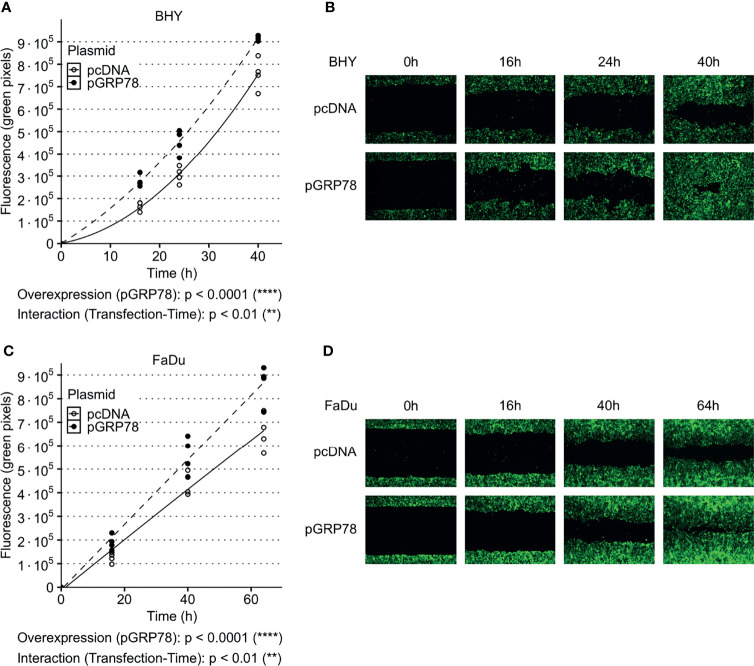
Increased migration of HNSCC cells after overexpression of GRP78. **(A–D)** Cell migration analysis (gap filling) of GFP-expressing BHY **(A, B)** and FaDu **(C, D)** cells. Both cell lines show an increase in migration after overexpression of GRP78 in comparison to control. Cells were transfected with GRP78 plasmid (pGRP78) 72 h prior to migration analysis. Cells transfected with an empty vector (pcDNA) were used as control (n = 4, Two-way RM ANOVA).

Both GRP78-overexpressing cell lines also showed a significant increase in their ability to migrate into the cell-free gaps compared to vector control cells (**Figures 4A–D**). Cell viability was not affected by overexpression between 24 and 96 h compared to control transfection, with the 24 h timepoint being the start of cell migration assay (**Supplementary Figure S3A, B**).

### Inhibition of GRP78 Leads to Decreased Migration in HNSCC Cells

**Figure 5:**
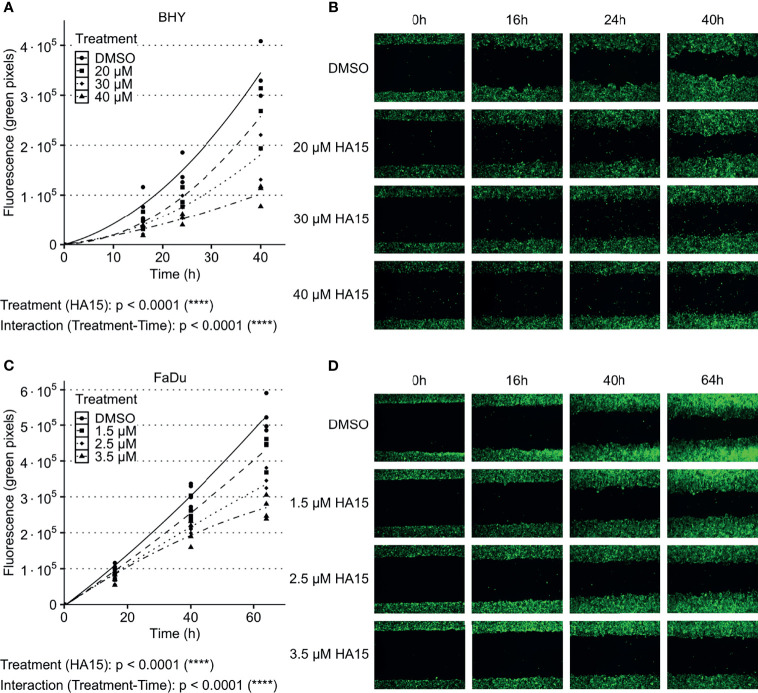
Inhibition of GRP78 impairs migration in HNSCC cells. **(A–D)** Cell migration analysis (gap filling) of GFP-expressing BHY **(A, B)** and FaDu **(C, D)** cells after treatment with GRP78-inhibitor HA15 in different concentrations show a dose dependent decrease of cell migration. DMSO was used as control (n ≤ 4, Two-way RM ANOVA).

The GRP78 inhibitor HA15 was used to block GRP78 function in BHY and FaDu cells. Cell viability analysis showed no significant effect of inhibitor treatment for both cell lines in comparison to control treatment with DMSO (**Supplementary Figures S3C, D**). Analysis of cell migration showed a dose-dependent decrease in cell motility in both cell lines in comparison to DMSO control treatment (**Figures 5A–D**). An analysis of the radiosensitivity was not possible as the drug treatment proved toxic due to low cell numbers and extended culture times needed for colony growth.

### Radiation Increased GRP78 Levels in HNSCC-Derived EVs

EVs derived from irradiated HNSCC donor cells have previously shown changes in their cargo and their ability to increase both the migratory potential and radioresistance of recipient cells (17, 19).

**Figure 6:**
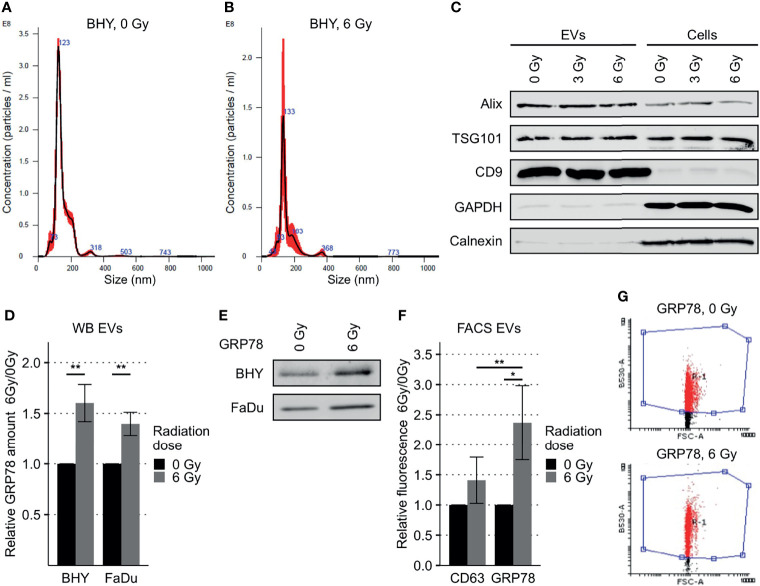
Increased GRP78 expression in HNSCC EVs. **(A, B)** Nanoparticle tracking analysis of EVs collected *via* serial ultracentrifugation from cell culture supernatant of irradiated (6 Gy) and non-irradiated (0Gy) BHY cells. **(C)** Western Blot of BHY EVs with positive (Alix, TSG101, CD9) and negative (GAPDH, Calnexin) EV markers. **(D, E)** Western Blot analysis with representative blot **(E)** shows increased amounts of GRP78 in EVs derived from irradiated cells (6Gy-EVs) in comparison to EVs derived from non-irradiated HNSCC cells (0Gy-EVs). GRP78 expression was normalized to Ponceau S staining and 0 Gy served as baseline (n = 4, t-test, **p < 0.01). **(F, G)** Flow cytometry analysis of GRP78 surface expression on BHY EVs show increased GRP78 on 6Gy-EVs compared to 0Gy-EVs. CD63 was used as control surface antigen showing no significant change in radiation induced surface expression (n = 4, t-test, *p adj. < 0.05). **(G)** Example dot blots of bead-coupled EVs in flow cytometric analysis.

The identity of small EVs recovered from culture supernatants was confirmed by analysis of the particle size, showing a diameter of approximately 130 nm on average, the expression of the EV marker proteins Alix, TSG101 and CD9 and the absence of ER/cytosolic marker proteins Calnexin and GAPDH (**Figures 6A–C**). Analysis of total GRP78 content of EVs released from irradiated cells (6Gy-EVs) displayed greater amounts of GRP78 in comparison to EVs derived from the respective non-irradiated control cells (0Gy-EVs) (**Figures 6D, E** and **Supplementary Figures S1E, F**). Elevated levels of GRP78 were also found on the surface of 6Gy-EVs in comparison to 0Gy-EVs. Comparison of radiation-induced changes in GRP78 located on the EV surface showed a significantly greater change in expression of GRP78 relative to that of EV surface marker CD63. CD63 surface expression did not change significantly after donor cell irradiation (**Figures 6F, G**).

### EVs From Irradiated HNSCC Cells Increased GRP78 in Non-Irradiated Recipient Cells

**Figure 7:**
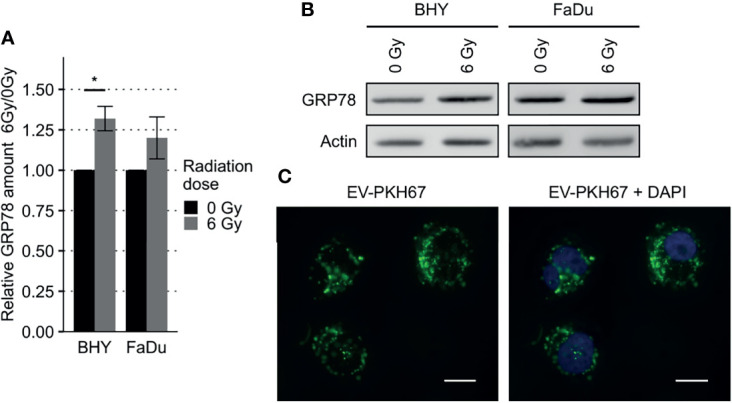
Increased GRP78 expression in HNSCC EV recipient cells. **(A, B)** Western Blot analysis with representative blot **(B)** of GRP78 expression in EV recipient cells show increased amounts of GRP78 in 6Gy-EV recipient cells. GRP78 expression was normalized to beta-actin and 0Gy-EV recipient cell GRP78 expression served as baseline (n = 3, t-test, *p < 0.05). **(C)** Fluorescence microscopy images of BHY cells co-cultured with PKH67-stained EVs documenting uptake. Stained vesicle membranes are shown in green. Recipient cell nuclei are shown in blue. White size bar indicates 20 µm.

To analyze the effect of EVs derived from irradiated (6Gy-EVs) and non-irradiated HNSCC cells (0Gy-EVs) on the GRP78 expression of non-irradiated recipient cells, both cell lines were co-cultivated with the respective EVs. The addition of 6Gy-EVs onto non-irradiated recipient BHY or FaDu cells led to these cells showing increased amounts of GRP78 in comparison to 0Gy-EVs (**Figures 7A, B**). To verify that isolated EVs were incorporated by recipient cells, 0Gy-EVs were stained with fluorescent membrane dye PKH67, co-cultured with recipient cells and analyzed with fluorescence microscopy (**Figure 7C**).

## Discussion

Increases in radioresistance and enhanced invasive or metastatic properties are major difficulties encountered during the radiotherapy of HNSCC (3, 31, 32). We now show an association between higher levels of GRP78 gene expression in HNSCC tumor tissues and a poorer therapy outcome for patients, indicating that GRP78 may play an important role in the response of HNSCC to treatment. Our *in vitro* analyses confirmed GRP78 as a component of EVs as well as driver of migration and radioresistance. Consequently, GRP78 may promote cancer radioresistance and cell motility during radiotherapy in irradiated cells and non-irradiated bystander cells.

In line with our findings, an association between poor treatment outcome and GRP78 overexpression was also found in various other tumor types including pancreatic cancer (33), ureter cancer (34), and esophageal carcinoma (35).

*In vitro* expression analysis showed increases in surface GRP78 in FaDu and BHY cells after irradiation, while intracellular levels were not affected. This suggests that radiation induces a combination of GRP78 overexpression and relocalization to the cell surface. Surface GRP78 was previously shown to influence migration and invasion in other cancer types. In colorectal and pancreatic cancer, surface GRP78 was shown to induce MMP and FAK activity promoting migration and invasion (36, 37). In prostate and pancreatic cancer, surface GRP78 triggered Akt signaling, thereby inducing migration and blocking apoptosis (5, 38–40). Accordingly, a knockout of GRP78 suppressed Akt activation *in vivo* (5). In esophageal carcinoma, a GRP78 knockdown decreased cell migration and invasion (35). Another study showed an increase in migration after irradiation in HNSCC cells (41). In line with these observations, we found increased migration in HNSCC cells after overexpression of GRP78, while blocking by a small molecule inhibitor reduced migration.

Beside increased cell migration, overexpression of GRP78 resulted in increased radioresistance in BHY and FaDu cells. In accord with these findings, cell viability after irradiation was reduced by a surface GRP78 blocking peptide upregulating apoptosis in pancreatic cancer (40). In oropharyngeal cancer, radioresistance was impaired by GRP78 silencing which reduced double-strand break repair and increased radiation-induced apoptosis (42). In another study on pancreatic cancer, a knockdown of GRP78 also reduced DNA repair visualized by γH2AX and 53BP1 foci (43). In lung cancer and glioblastoma, a GRP78 antibody enhanced radiosensitivity by upregulation of apoptosis *in vitro* and the combined antibody/radiation treatment was the most effective treatment for *in vivo* models for both tumor types (44).

Other important factors of malignancy in HNSCC are non-targeted effects of ionizing radiation, which occur in non-irradiated, neighboring, and distant cells (45, 46). Besides cell-cell signaling *via* gap junctions and the release of soluble factors (45) EV signaling is also associated with non-targeted effects (47), which may induce metastases as well as resistance to drugs and radiation (2, 18, 48). EVs are thought to influence therapy outcome by altering adjacent and even distant non-irradiated cells after radiotherapy (49). For example, EVs derived from irradiated glioblastoma cells showed an altered composition and conferred increased FAK activation, migratory potential and radioresistance to recipient cells (50, 51). Surface HSP70-positive EVs derived from pancreas and colon carcinoma cells could increase the migratory potential of NK cells (52). In neuroblastoma cells, increased radioresistance could be observed in recipient cells co-cultured with EVs isolated from irradiated cells (53). Moreover, reduced EV surface levels of another chaperone, the heat shock protein HSP70, have been correlated with a better prognosis and therapy response in some cancer types (54, 55).

Similar results could be found in our previous studies which showed that EVs derived from irradiated HNSCC cells contained a different protein cargo with an increased amount of migration related molecules. Those vesicles activated the recipient cell Akt pathway, MMP activity and subsequently promoted migration (17). Moreover, the same vesicles increased the radioresistance of recipient cells *via* upregulation of double-strand break repair mechanisms observable by a more rapid decrease in 53BP1 foci after irradiation (19). Here, it was shown that irradiation increased GRP78 expression on the EV surface. Furthermore, increased GRP78 levels could be conferred to recipient cells when they were co-cultivated with the GRP78-rich vesicles from irradiated cells, indicating the chaperone may have a hitherto unrecognized action in systemic (non-targeted) effects of local irradiation.

In summary, this study, together with our previous data (17, 19), suggests a model of increased radioresistance and cell motility, conferred directly and indirectly by the radiation-induced overexpression of cell surface GRP78. Specifically, surface GRP78 can either support radioresistance and migration in irradiated cells or be transported *via* EVs to non-irradiated tumor recipient cells. Based on the similar activities of GRP78 and EVs from irradiated cells it seems that EV-mediated transfer of GRP78 may be a vital component in radioresistance and migration of HNSCC cells. Given that GRP78 surface localization seems to be a tumor cell specific process, absent in non-malignant cells (12, 55), targeting GRP78 and/or EV-mediated GRP78 transfer might be attractive for future therapeutic interventions in radiotherapy of HNSCC.

## Data Availability Statement

The original contributions presented in the study are included in the article/**Supplementary Material**. Further inquiries can be directed to the corresponding author.

## Author Contributions

SM, MS, MA, and MP contributed to conception and design of the study. MS, KW, and RK performed experiments. MS and SM performed formal data analysis and the statistical calculations. MS wrote the first draft of the manuscript. SM was responsible for project administration and supervision. All authors contributed to manuscript revision, read, and approved the submitted version.

## Conflict of Interest

The authors declare that the research was conducted in the absence of any commercial or financial relationships that could be construed as a potential conflict of interest.

The reviewer GM declared a shared parent affiliation with the authors MA and MP to the handling editor at the time of review.

## Publisher’s Note

All claims expressed in this article are solely those of the authors and do not necessarily represent those of their affiliated organizations, or those of the publisher, the editors and the reviewers. Any product that may be evaluated in this article, or claim that may be made by its manufacturer, is not guaranteed or endorsed by the publisher.

## Abbreviations

EV, Extracellular vesicle; EdFBS, EV-depleted FBS; GRP78, Glucose-regulated protein 78kDa; HNSCC, Head and neck squamous cell carcinoma; SF2, survival fraction after 2-Gy irradiation; UPR, Unfolded protein response.

## References

[1] Chow LQM. Head and Neck Cancer. N Engl J Med (2020) 382(1):60–72. doi: 10.1056/NEJMra1715715 31893516

[2] Xie C Ji N Tang Z Li J Chen Q. The Role of Extracellular Vesicles From Different Origin in the Microenvironment of Head and Neck Cancers. Mol Cancer (2019) 18(1):83. doi: 10.1186/s12943-019-0985-3 30954079 PMC6451295

[3] Alsahafi E Begg K Amelio I Raulf N Lucarelli P Sauter T. Clinical Update on Head and Neck Cancer: Molecular Biology and Ongoing Challenges. Cell Death Dis (2019) 10(8):540. doi: 10.1038/s41419-019-1769-9 31308358 PMC6629629

[4] Durante M Loeffler JS. Charged Particles in Radiation Oncology. Nat Rev Clin Oncol (2010) 7(1):37–43. doi: 10.1038/nrclinonc.2009.183 19949433

[5] Fu Y Wey S Wang M Ye R Liao CP Roy-Burman P. Pten Null Prostate Tumorigenesis and AKT Activation Are Blocked by Targeted Knockout of ER Chaperone GRP78/BiP in Prostate Epithelium. Proc Natl Acad Sci USA (2008) 105(49):19444–9. doi: 10.1073/pnas.0807691105 PMC2614780 19033462

[6] Tsai Y-L Zhang Y Tseng C-C Stanciauskas R Pinaud F Lee AS. Characterization and Mechanism of Stress-Induced Translocation of 78-Kilodalton Glucose-Regulated Protein (GRP78) to the Cell Surface. J Biol Chem (2015) 290(13):8049–64. doi: 10.1074/jbc.M114.618736 PMC4375463 25673690

[7] Cole DW Svider PF Shenouda KG Lee PB Yoo NG McLeod TM. Targeting the Unfolded Protein Response in Head and Neck and Oral Cavity Cancers. Exp Cell Res (2019) 382(1):111386. doi: 10.1016/j.yexcr.2019.04.007 31075256 PMC6867800

[8] Wang M Wey S Zhang Y Ye R Lee AS. Role of the Unfolded Protein Response Regulator GRP78/BiP in Development, Cancer, and Neurological Disorders. Antioxid Redox Signal (2009) 11(9):2307–16. doi: 10.1089/ars.2009.2485 PMC2819800 19309259

[9] Farshbaf M Khosrowshahi AY Mojarad-Jabali S Zarebkohan A Valizadeh H Walker PR. Cell Surface GRP78: An Emerging Imaging Marker and Therapeutic Target for Cancer. J Control Release (2020) 932–41. doi: 10.1016/j.jconrel.2020.10.055 33129921

[10] Ni M Zhang Y Lee AS. Beyond the Endoplasmic Reticulum: Atypical GRP78 in Cell Viability, Signalling and Therapeutic Targeting. Biochem J (2011) 434(2):181–8. doi: 10.1042/BJ20101569 PMC3353658 21309747

[11] Li N Zoubeidi A Beraldi E Gleave ME. GRP78 Regulates Clusterin Stability, Retrotranslocation and Mitochondrial Localization Under ER Stress in Prostate Cancer. Oncogene (2013) 32(15):1933–42. doi: 10.1038/onc.2012.212 22689054

[12] Zhang Y Tseng C-C Tsai Y-L Fu X Schiff R Lee AS. Cancer Cells Resistant to Therapy Promote Cell Surface Relocalization of GRP78 Which Complexes With PI3K and Enhances PI (3, 4, 5) P3 Production. PloS One (2013) 8(11):e80071. doi: 10.1371/journal.pone.0080071 24244613 PMC3823711

[13] Lu G Luo H Zhu X. Targeting the GRP78 Pathway for Cancer Therapy. Front Med (2020) 7:351. doi: 10.3389/fmed.2020.00351 PMC7409388 32850882

[14] Yao X Liu H Zhang X Zhang L Li X Wang C. Cell Surface GRP78 Accelerated Breast Cancer Cell Proliferation and Migration by Activating STAT3. PloS One (2015) 10(5):e0125634. doi: 10.1371/journal.pone.0125634 25973748 PMC4431800

[15] Liu R Li X Gao W Zhou Y Wey S Mitra SK. Monoclonal Antibody Against Cell Surface GRP78 as a Novel Agent in Suppressing PI3K/AKT Signaling, Tumor Growth, and Metastasis. Clin Cancer Res (2013) 19(24):6802–11. doi: 10.1158/1078-0432.CCR-13-1106 PMC4151476 24048331

[16] Xiao D Ohlendorf J Chen Y Taylor DD Rai SN Waigel S. Identifying mRNA, microRNA and Protein Profiles of Melanoma Exosomes. PloS One (2012) 7(10):e46874. doi: 10.1371/journal.pone.0046874 23056502 PMC3467276

[17] Mutschelknaus L Azimzadeh O Heider T Winkler K Vetter M Kell R. Radiation Alters the Cargo of Exosomes Released From Squamous Head and Neck Cancer Cells to Promote Migration of Recipient Cells. Sci Rep (2017) 7(1):12423. doi: 10.1038/s41598-017-12403-6 28963552 PMC5622080

[18] Wang X Guo J Yu P Guo L Mao X Wang J. The Roles of Extracellular Vesicles in the Development, Microenvironment, Anticancer Drug Resistance, and Therapy of Head and Neck Squamous Cell Carcinoma. J Exp Clin Cancer Res (2021) 40(1):35. doi: 10.1186/s13046-021-01840-x 33478586 PMC7819156

[19] Mutschelknaus L Peters C Winkler K Yentrapalli R Heider T Atkinson MJ. Exosomes Derived From Squamous Head and Neck Cancer Promote Cell Survival After Ionizing Radiation. PloS One (2016) 11(3):e0152213. doi: 10.1371/ journal.pone.0152213 27006994 10.1371/journal.pone.0152213 PMC4805173

[20] Mounir M Lucchetta M Silva TC Olsen C Bontempi G Chen X. New Functionalities in the TCGAbiolinks Package for the Study and Integration of Cancer Data From GDC and GTEx. PloS Comput Biol (2019) 15(3):e1006701. doi: 10.1371/journal.pcbi.1006701 30835723 PMC6420023

[21] Silva TC Colaprico A Olsen C D'Angelo F Bontempi G Ceccarelli M. Analyze Cancer Genomics and Epigenomics Data Using Bioconductor Packages. F1000Res (2016) 5:1542. doi: 10.12688/f1000research.8923.2 28232861 PMC5302158

[22] Colaprico A Silva TC Olsen C Garofano L Cava C Garolini D. TCGAbiolinks: An R/Bioconductor Package for Integrative Analysis of TCGA Data. Nucleic Acids Res (2016) 44(8):e71. doi: 10.1093/nar/gkv1507 26704973 PMC4856967

[23] Ritchie ME Phipson B Wu D Hu Y Law CW Shi W. Limma Powers Differential Expression Analyses for RNA-Sequencing and Microarray Studies. Nucleic Acids Res (2015) 43(7):e47. doi: 10.1093/nar/gkv007 25605792 PMC4402510

[24] McCarthy DJ Chen Y Smyth GK. Differential Expression Analysis of Multifactor RNA-Seq Experiments With Respect to Biological Variation. Nucleic Acids Res (2012) 40(10):4288–97. doi: 10.1093/nar/gks042 PMC3378882 22287627

[25] Goldman MJ Craft B Hastie M Repečka K McDade F Kamath A. Visualizing and Interpreting Cancer Genomics Data *via* the Xena Platform. Nat Biotechnol (2020) 38(6):675–8. doi: 10.1038/s41587-020-0546-8 PMC7386072 32444850

[26] Weller H. Countcolors: Locates and Counts Pixels Within Color Range(s) in Images (2019). Available at: https://CRANR-projectorg/package=countcolors.

[27] Team RC. R: A Language and Environment for Statistical Computing. R Foundation for Statistical Computing. Vienna, Austria: R Foundation for Statistical Computing (2021). Available at: https://wwwR-projectorg.

[28] Team R. RStudio: Integrated Development for R. Boston, MA: RStudio, PBC (2021). Available at: http://wwwrstudiocom.

[29] Wickham H. Ggplot2: Elegant Graphics for Data Analysis. Springer-Verlag New York (2009).

[30] Kassambara A. Rstatix: Pipe-Friendly Framework for Basic Statistical Tests (2021). Available at: https://CRANR-projectorg/package=rstatix.

[31] Vilalta M Rafat M Graves EE. Effects of Radiation on Metastasis and Tumor Cell Migration. Cell Mol Life Sci (2016) 73(16):2999–3007. doi: 10.1007/s00018-016-2210-5 27022944 PMC4956569

[32] Sundahl N Duprez F Ost P De Neve W Mareel M. Effects of Radiation on the Metastatic Process. Mol Med (2018) 24(1):16. doi: 10.1186/s10020-018-0015-8 30134800 PMC6016893

[33] Niu Z Wang M Zhou L Yao L Liao Q Zhao Y. Elevated GRP78 Expression Is Associated With Poor Prognosis in Patients With Pancreatic Cancer. Sci Rep (2015) 5:16067. doi: 10.1038/srep16067 26530532 PMC4632002

[34] Park CH Choi MS Ha JY Kim BH Kim CI. Effect of Overexpression of Glucose-Regulated Protein 78 and Bcl-2 on Recurrence and Survival in Patients With Ureter Tumors. Korean J Urol (2013) 54(10):671–6. doi: 10.4111/kju.2013.54.10.671 PMC3806990 24175040

[35] Ren P Chen C Yue J Zhang J Yu Z. High Expression of Glucose-Regulated Protein 78 (GRP78) Is Associated With Metastasis and Poor Prognosis in Patients With Esophageal Squamous Cell Carcinoma. Oncol Targets Ther (2017) 10:617–25. doi: 10.2147/OTT.S123494 PMC5312696 28228658

[36] Li Z Zhang L Zhao Y Li H Xiao H Fu R. Cell-Surface GRP78 Facilitates Colorectal Cancer Cell Migration and Invasion. Int J Biochem Cell Biol (2013) 45(5):987–94. doi: 10.1016/j.biocel.2013.02.002 23485528

[37] Yuan XP Dong M Li X Zhou JP. GRP78 Promotes the Invasion of Pancreatic Cancer Cells by FAK and JNK. Mol Cell Biochem (2015) 398(1-2):55–62. doi: 10.1007/s11010-014-2204-2 25218495

[38] Misra UK Deedwania R Pizzo SV. Activation and Cross-Talk Between Akt, NF-Kappab, and Unfolded Protein Response Signaling in 1-LN Prostate Cancer Cells Consequent to Ligation of Cell Surface-Associated GRP78. J Biol Chem (2006) 281(19):13694–707. doi: 10.1074/jbc.M511694200 16543232

[39] Misra UK Deedwania R Pizzo SV. Binding of Activated Alpha2-Macroglobulin to Its Cell Surface Receptor GRP78 in 1-LN Prostate Cancer Cells Regulates PAK-2-Dependent Activation of LIMK. J Biol Chem (2005) 280(28):26278–86. doi: 10.1074/jbc.M414467200 PMC1201553 15908432

[40] Gopal U Mowery Y Young K Pizzo SV. Targeting Cell Surface GRP78 Enhances Pancreatic Cancer Radiosensitivity Through YAP/TAZ Protein Signaling. J Biol Chem (2019) 294(38):13939–52. doi: 10.1074/jbc.RA119.009091 PMC6755808 31358620

[41] Pickhard AC Margraf J Knopf A Stark T Piontek G Beck C. Inhibition of Radiation Induced Migration of Human Head and Neck Squamous Cell Carcinoma Cells by Blocking of EGF Receptor Pathways. BMC Cancer (2011) 11:388. doi: 10.1186/1471-2407-11-388 21896192 PMC3224383

[42] Sun C Han C Jiang Y Han N Zhang M Li G. Inhibition of GRP78 Abrogates Radioresistance in Oropharyngeal Carcinoma Cells After EGFR Inhibition by Cetuximab. PloS One (2017) 12(12):e0188932. 29232380 10.1371/journal.pone.0188932 PMC5726659

[43] Dauer P Sharma NS Gupta VK Durden B Hadad R Banerjee S. ER Stress Sensor, Glucose Regulatory Protein 78 (GRP78) Regulates Redox Status in Pancreatic Cancer Thereby Maintaining “Stemness”. Cell Death Dis (2019) 10(2):1–13. doi: 10.1038/s41419-019-1408-5 PMC6372649 30755605

[44] Dadey DYA Kapoor V Hoye K Khudanyan A Collins A Thotala D. Antibody Targeting GRP78 Enhances the Efficacy of Radiation Therapy in Human Glioblastoma and Non–Small Cell Lung Cancer Cell Lines and Tumor Models. Clin Cancer Res (2017) 23(10):2556–64. doi: 10.1158/1078-0432.CCR-16-1935 27815359

[45] Ladjohounlou R Louati S Lauret A Gauthier A Ardail D Magne N. Ceramide-Enriched Membrane Domains Contribute to Targeted and Nontargeted Effects of Radiation Through Modulation of PI3K/AKT Signaling in HNSCC Cells. Int J Mol Sci (2020) 21(19):7200. doi: 10.3390/ijms21197200 PMC7582380 33003449

[46] Blyth BJ Sykes PJ. Radiation-Induced Bystander Effects: What Are They, and How Relevant Are They to Human Radiation Exposures? Radiat Res (2011) 176(2):139–57. doi: 10.1667/RR2548.1 21631286

[47] Al-Mayah A Bright S Chapman K Irons S Luo P Carter D. The Non-Targeted Effects of Radiation Are Perpetuated by Exosomes. Mutat Res/Fundamental Mol Mech Mutagenesis (2015) 772:38–45. 10.1016/j.mrfmmm.2014.12.007 25772109

[48] Sakha S Muramatsu T Ueda K Inazawa J. Exosomal microRNA miR-1246 Induces Cell Motility and Invasion Through the Regulation of DENND2D in Oral Squamous Cell Carcinoma. Sci Rep (2016) 6(1):1–11. doi: 10.1038/srep38750 27929118 PMC5144099

[49] Jabbari N Karimipour M Khaksar M Akbariazar E Heidarzadeh M Mojarad B. Tumor-Derived Extracellular Vesicles: Insights Into Bystander Effects of Exosomes After Irradiation. Lasers Med Sci (2020) 35(3):531–45. doi: 10.1007/s10103-019-02880-8 31529349

[50] Arscott WT Tandle AT Zhao S Shabason JE Gordon IK Schlaff CD. Ionizing Radiation and Glioblastoma Exosomes: Implications in Tumor Biology and Cell Migration. Transl Oncol (2013) 6(6):638–48. doi: 10.1593/tlo.13640 PMC3890698 24466366

[51] Mrowczynski OD Madhankumar AB Sundstrom JM Zhao Y Kawasawa YI Slagle-Webb B. Exosomes Impact Survival to Radiation Exposure in Cell Line Models of Nervous System Cancer. Oncotarget (2018) 9(90):36083–101. doi: 10.18632/oncotarget.26300 PMC6281426 30546829

[52] Gastpar R Gehrmann M Bausero MA Asea A Gross C Schroeder JA. Heat Shock Protein 70 Surface-Positive Tumor Exosomes Stimulate Migratory and Cytolytic Activity of Natural Killer Cells. Cancer Res (2005) 65(12):5238–47. doi: 10.1158/0008-5472.CAN-04-3804 PMC1785299 15958569

[53] Cerreto M Sennato S Tortolici F Casciardi S Giovanetti A Rufini S. Effect of the Irradiation on Neuroblastoma-Derived Microvesicles: A Physical and Biological Investigation. Colloids Surf A (2017) 109–202. doi: 10.1016/j.colsurfa.2017.05.029

[54] Chanteloup G Cordonnier M Isambert N Bertaut A Hervieu A Hennequin A. Monitoring HSP70 Exosomes in Cancer Patients' Follow Up: A Clinical Prospective Pilot Study. J Extracell Vesicles (2020) 9(1):1766192. doi: 10.1080/20013078.2020.1766192 32595915 PMC7301715

[55] Gobbo J Marcion G Cordonnier M Dias AMM Pernet N Hammann A. Restoring Anticancer Immune Response by Targeting Tumor-Derived Exosomes With a HSP70 Peptide Aptamer. J Natl Cancer Inst (2016) 108(3). doi: 10.1093/jnci/djv330 26598503

