# Supplementary material for: The Chaperone Protein GRP78 Promotes Survival and Migration of Head and Neck Cancer After Direct Radiation Exposure and Extracellular Vesicle-Transfer
Source: Front Oncol. 2022 Mar 1;12:842418. doi: 10.3389/fonc.2022.842418 (PMC8921984; doi:10.3389/fonc.2022.842418)
Supplement: Supplementary file 1 [file DataSheet_1.docx]

Supplementary Material

##
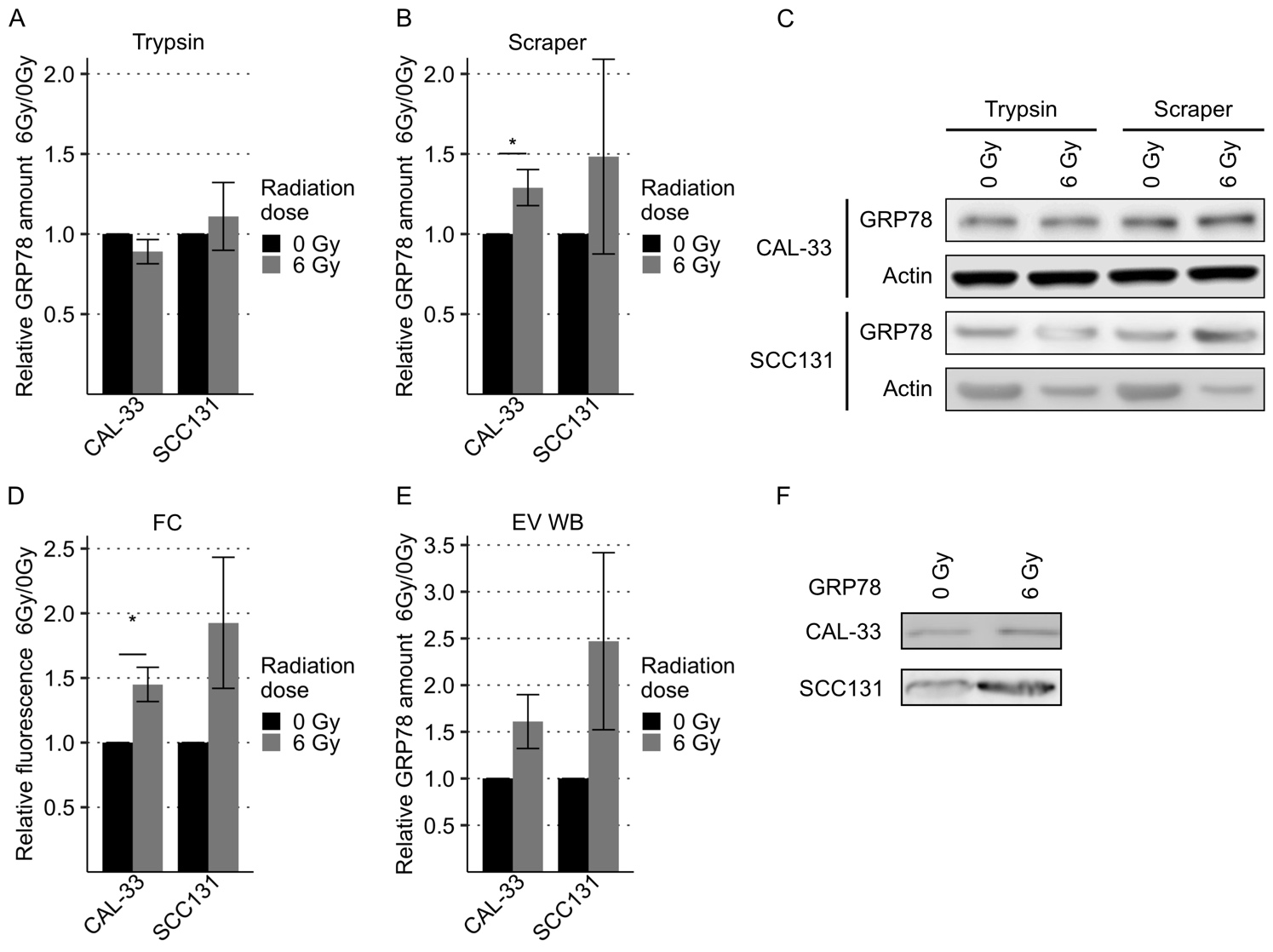


**Supplementary Figure 1**. Increased GRP78 expression on the cell surface of additional HNSCC cell lines after irradiation and in EVs derived from respective cells. **(A-C)** Western Blot analysis of GRP78 expression in non-irradiated (0 Gy) and irradiated (6 Gy) HNSCC cells harvested with trypsin show no significant difference **(A).** Cells harvested with a cell scraper show an increase in GRP78 after irradiation **(B)**. GRP78 expression was normalized to beta-actin and 0 Gy samples served as baseline (n = 3, t-test, * p < 0.05). **(C)** Representative Western Blot images of GRP78 expression in CAL-33 and SCC131 cells. Beta-actin was used as loading control. **(D)** Flow cytometry (FC) analysis of surface GRP78 in 0 Gy and 6 Gy-irradiated HNSCC cells confirmed increased GRP78 expression after irradiation. Samples were normalized to secondary antibody signal and 0 Gy (n = 3, t-test, * p < 0.05). **(E, F)** Western Blot analysis (EV WB) with representative blot **(F)** shows increased amounts of GRP78 in EVs derived from irradiated cells (6 Gy) in comparison to EVs derived from non-irradiated HNSCC cells (0 Gy). GRP78 expression was normalized to Ponceau S staining and 0 Gy served as baseline (n = 3, t-test).


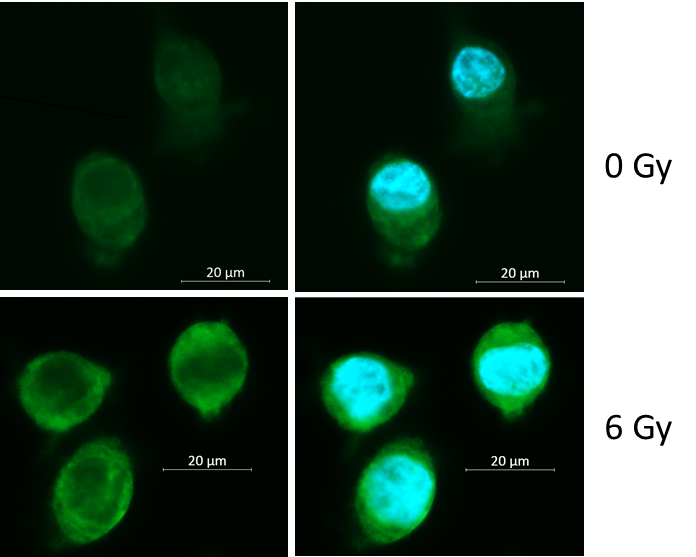


**Supplementary Figure 2.** Fluorescence microscopy images of BHY cells 24 h after irradiation with 0 and 6 Gy. Cells were fixed in 4 % paraformaldehyde without permeabilization for appropriate labelling of surface GRP78 and stained with an anti-GRP78 antibody. GRP78 expression is shown in green, cell nuclei are shown in blue. White size bar indicates 20 µm.

**Supplementary Figure 3.** Viability of BHY and FaDu cells after treatment with GRP78-inhibitor or overexpression of GRP78. **(A, B)** Cell viability assay of BHY **(A)** and **(B)** FaDu cells show no significant differences in cell viability 24 to 96 h after lipofectamine-mediated overexpression of GRP78 (pGRP78). Vector pcDNA was used as control transfection (n = 3, pairwise t-test). **(C, D)** Cell viability assay of BHY **(C)** and **(D)** FaDu cells after treatment with different concentrations of the GRP78 inhibitor HA15 for up to 72h show no significant differences in cell viability. Values were normalized to the DMSO control (n = 3, pairwise t-test).
